# Beneficial Effects of Natural Mineral Waters on Intestinal Inflammation and the Mucosa-Associated Microbiota

**DOI:** 10.3390/ijms22094336

**Published:** 2021-04-21

**Authors:** Nicolas Barnich, Michael Rodrigues, Pierre Sauvanet, Caroline Chevarin, Sylvain Denis, Olivier Le Goff, Danielle Faure-Imbert, Thierry Hanh, Christian F Roques, Benoit Chassaing, Monique Alric

**Affiliations:** 1 Inserm U1071, USC-INRAE 2018, Microbes, Intestin, Inflammation et Susceptibilité de l’Hôte (M2iSH), Centre de Recherche en Nutrition Humaine Auvergne, Université Clermont Auvergne, 63001 Clermont-Ferrand, France; Michael.rodrigues@uca.fr (M.R.); pierre.sauvanet@uca.fr (P.S.); caroline.chevarin@uca.fr (C.C.); 2 Chirurgie Digestive, Centre Hospitalier Universitaire, 63000 Clermont-Ferrand, France; 3 UMR UCA-INRAE 454 MEDIS, Microbiology, Digestive Environment and Health, Centre de Recherche en Nutrition Humaine-Auvergne, Université Clermont Auvergne, 63001 Clermont-Ferrand, France; sylvain.denis@uca.fr (S.D.); olivierlegoff2@gmail.com (O.L.G.); Monique.alric@uca.fr (M.A.); 4 S.E.M. Chatel-Développement, 63140 Châtel-Guyon, France; dfaureimbert@wanadoo.fr; 5 HAT Consultant, 75006 Paris, France; thierry.hanh@gmail.com; 6 Department of Physical & Rehabilitation Medicine, Toulouse III University, 31400 Toulouse, France; cf.roques@gmail.com; 7 Inserm U1016, Team ‘‘Mucosal Microbiota in Chronic Inflammatory Diseases’’, CNRS UMR 8104, Université de Paris, 75014 Paris, France; benoit.chassaing@inserm.fr

**Keywords:** natural mineral water, intestinal inflammation, mucosa-associated microbiota

## Abstract

Natural mineral water (NMWs) intake has been traditionally used in the treatment of various gastrointestinal diseases. We investigated the effect of two French NMWs, one a calcium and magnesium sulphate, sodium chloride, carbonic, and ferruginous water (NMW1), the other a mainly bicarbonate water (NMW2) on the prevention of intestinal inflammation. Intestinal epithelial cells stimulated with heat inactivated *Escherichia coli* or H_2_O_2_ were treated with NMWs to evaluate the anti-inflammatory effects. Moderate colitis was induced by 1% dextran sulfate sodium (DSS) in Balbc/J mice drinking NMW1, NWW2, or control water. General signs and histological features of colitis, fecal lipocalin-2 and pro-inflammatory KC cytokine levels, global mucosa-associated microbiota, were analyzed. We demonstrated that both NMW1 and NMW2 exhibited anti-inflammatory effects using intestinal cells. In induced-colitis mice, NMW1 was effective in dampening intestinal inflammation, with significant reductions in disease activity scores, fecal lipocalin-2 levels, pro-inflammatory KC cytokine release, and intestinal epithelial lesion sizes. Moreover, NMW1 was sufficient to prevent alterations in the mucosa-associated microbiota. These observations, through mechanisms involving modulation of the mucosa-associated microbiota, emphasize the need of investigation of the potential clinical efficiency of such NMWs to contribute, in human beings, to a state of low inflammation in inflammatory bowel disease.

## 1. Introduction

The beneficial effect of treatments using natural mineral waters (NMW) has been recognized for decades with water from several resorts in France and Europe recommended for various diseases of the gastrointestinal tract, such as gastric dyspepsia [1,2,3] and irritable bowel syndrome [2,4]. The spa doctors’ clinical experience investigates also the interest of such thermo–mineral treatments for some aspects of inflammatory bowel disease (IBD) and also observes an improvement of digestive symptoms in patients with IBD spondylitis undergoing rheumatological spa treatment [5]. In French spa care facilities, NMW are used for various treatments through topical, oral, or rectal (enteroclysis) administration. Enteroclysis, an essential element of the thermo–mineral treatment for colitis, is made up of a rectal drip but also NMW drinking and abdominal application.

A major difficulty in evaluating the effects of this treatment is the absence of specific biological markers of the pathologies evaluated, especially functional pathologies, and the absence of clear pathophysiological explanations of the causes of these diseases. These issues explain the difficulty in demonstrating objective changes in biological parameters specific to the conditions considered following spa treatment. Recently, a series of studies concerning the involvement of the intestinal microbiota in the physiopathology of chronic diseases have been reported [6]. The microorganisms that live in the intestine, approximately 100,000 billion bacteria, are involved in many essential intestinal functions, including protective, digestive, and immune functions [7]. Many diseases of the gastrointestinal tract, both functional (e.g., irritable bowel syndrome) and organic (e.g., chronic inflammatory bowel diseases), are characterized by changes in the microbiota composition. Three groups or phyla (including over 500 species) dominate the human microbiota: *Bacteroidetes*, *Firmicutes,* and *Actinobacteria*. Different variations in their quantitative representation have been associated with gastrointestinal diseases. Thus, optimal clinical use of microbial manipulation may be used as an adjuvant to immunosuppressive therapy to accelerate and improve the induction of deep remission based on an individual patient’s microbial profile [8]. With knowledge of the metagenome of the human intestinal microbiota, it is now possible to monitor microbiota variations following spa treatment.

The evaluation of the intestinal microbiota in patients with IBD has identified generalized or localized dysbiosis, which corresponds to both a decrease in the number of usual bacteria and an increase in the number of unusual bacteria, with overall reductions in diversity and richness [9]. Gut microbiota of Crohn’s disease (CD) patients is different from the intestinal microbiota of patients with ulcerative colitis and controls [10]. Clinical and experimental evidence show that the intestinal microbiota exerts deleterious pro-inflammatory effects in CD. Several independent teams studied the involvement of the microbiota in the post-operative recurrence of CD by different approaches. First, the presence of a particular *Escherichia coli* pathovar with properties of invasion and adherence (AIEC) to the intestinal mucosa was reported [11], and on the other hand, the approaches of global microbiota studies have revealed many beneficial species, such as *Faecalibacterium prausnitzii* [12].

Natural mineral waters containing carbonic metabolites and/or sulphates and/or iron exhibited anti-inflammatory properties unobserved with tap water. The fundamental role of bicarbonates in vital systems was recently emphasized [13]. They are necessary to an efficient digestive tract protective barrier [14,15,16] and participate also in gut repair [17]. Sulphates reducing bacteria (SRB), most of them Proteobacteria (*Desulfovibrios*, *E. coli*), reduce, in the intestine, sulphate contained in food in an assimilatory way (ASR), leading to cysteine and methionine synthesis, or in a dissimilatory way (DSR) to produce energy with hydrogen sulfide liberation in the lumen [18,19]. This last compound is able to produce intestinal inflammatory damage [20,21] but can also show anti-inflammatory effects [22]. Sulphate waters could favor ASR reducing so SH2 production can occur in the lumen of the intestine and/or enhance the anti-inflammatory properties of SH2, both ways contributing to reduce local inflammation. Iron is widely involved in inflammation, tissue damage, or protection. The effects of iron supplementation on the gut could be beneficial through increasing local concentrations of anti-inflammatory fatty acids [23] and lowering inflammation [24]. Iron [25] and selenium [26] were shown to reduce inflammation in sulphate dextran colitis model. Thus, in the present study, we investigated the action of two NMW: NMW1 (a sulphate–carbonic–ferruginous water from the thermal springs of Châtel-Guyon, France) and NMW2 (mainly a bicarbonate water from the thermal springs in Vichy, France) to evaluate the actual beneficial effects of these NMW on intestinal inflammation.

## 2. Results

### 2.1. Inhibitory Effect of Mineral Waters on Induced Interleukin-8 (IL-8) Secretion by T84 Cells

**Figure 1:**
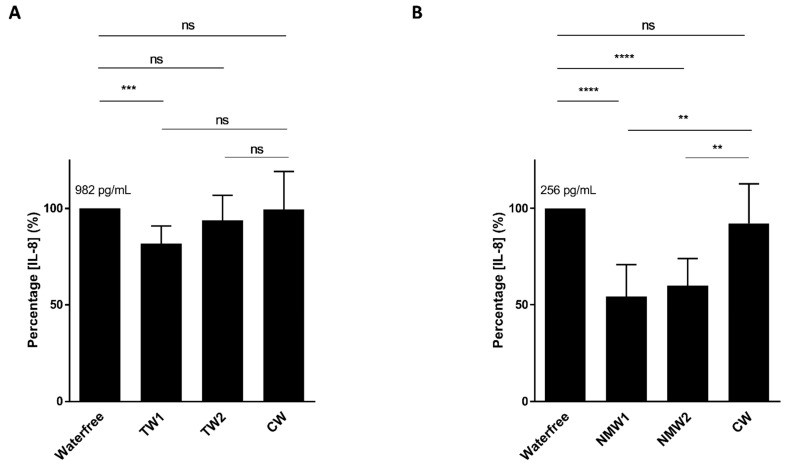
Secretion of the pro-inflammatory cytokine IL-8 by T84 cells after stimulation with an LF82 bacterial lysate (**A**) or exposure to H_2_O_2_ (**B**) in the presence of 50 µL of different waters (distilled water or NMW1 or NMW2). The results are presented as the mean ± SEM of at least five independent experiments. Statistical comparisons were carried out by one-way ANOVA with the Bonferroni post hoc test (** *p* < 0.01, *** *p* < 0.001, **** *p* < 0.0001, ns—not significant) after normality testing using the Kolmogorov–Smirnov test.

We first analyzed the abilities of thermal waters NMW1 and NMW2 to limit cytokine secretion by intestinal epithelial cells following bacterial stimulation or oxidative stress exposure. Following stimulation of T84 intestinal epithelial cells with an AIEC LF82 bacterial lysate and a 66-h period of treatment with 50 µL of thermal water, only the NMW1 led to significantly decreased production of the pro-inflammatory cytokine IL-8 compared to the NMW2 and control water (CW) (Figure 1A). Of note, similar data were obtained using 25 µL of NMW1 (data not shown). Following H_2_O_2_ stimulation, the use of NMW1 and NMW2 led to significant decreases in the secretion of the proinflammatory cytokine IL-8 by T84 cells compared to treatment with CW (Figure 1B). Of note, the level of IL-8 secretion from these same uninfected/unstimulated cells was 84 pg/mL. Together, the results demonstrate the anti-inflammatory properties of both NMW1 and NMW2.

### 2.2. Mineral Water Decreased the Severity of DSS-Induced Colitis

**Figure 2:**
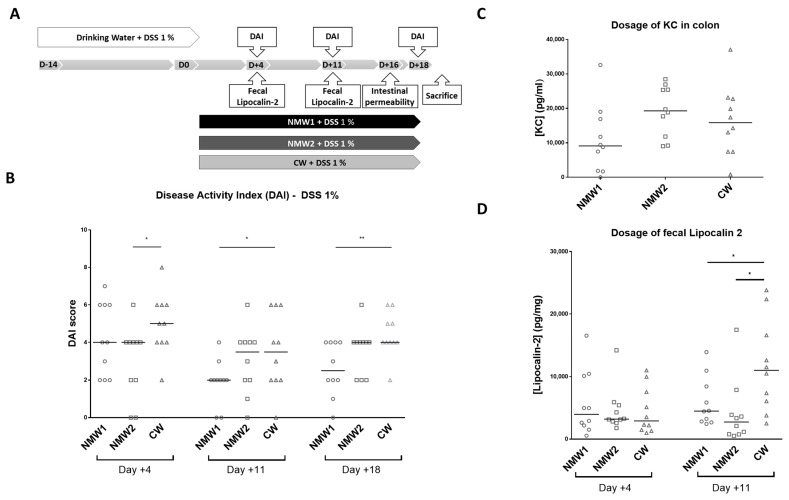
Effect of the administration of NMW1 or NMW2 on a DSS-induced inflammation model. (**A**) Experimental protocol. (**B**) Evaluation of the disease activity index (DAI) on days 4, 11, and 18. (**C**) Production of the proinflammatory cytokine KC by the colonic mucosa. (**D**) Determination of fecal lipocalin-2 levels. The results are presented as the median. Statistical comparisons were carried out after normality testing using Kolmogorov–Smirnov tests, and subsequent one-way ANOVA with the Bonferroni post hoc test was performed (* *p* < 0.05, ** *p* < 0.01).

We next evaluated the effects of NMW1 and NMW2 on a mouse model mimicking the inflammatory state observed in CD patients and compared the effects with those of CW. First, moderate colitis was induced in Balb/cJ mice via administration of 1% DSS in the drinking water, and the severity of colitis was evaluated with the disease activity index (DAI) following spa water administration for a 3-week period (Figure 2A). Interestingly, we observed that NMW1 significantly decreased the DAI score from day 11 until the end of the protocol (day 18) (Figure 2B) compared to NMW2 or CW. Of note, the DAI score of the animals not treated with DSS remained at 0 throughout the protocol (data not shown). DSS-treated mice receiving NMW1 had substantially improved intestinal permeability (Figure S1) and decreased secretion of the pro-inflammatory cytokine KC at the time of sacrifice (Keratinocyte Chemoattractant) (Figure 2C). To confirm the impact of thermal water on gut inflammation, we measured fecal Lcn-2 levels in mice that received or did not receive NMW. On day 11, the oral administration of either NMW1 or NMW2 led to decreased fecal Lcn-2 levels in mice treated with 1% DSS compared with oral administration of CW (Figure 2D). Together, these data demonstrated that both NMW1 and NMW2 were efficient and limited colitis after a 3-week treatment period, highlighting that a treatment with thermal water could limit the inflammatory environment in the gut. In a mouse model of severe colitis (4% DSS), no beneficial effect of treatment with NMW (NMW1 and NMW2) was observed (Figure S2).

**Figure 3:**
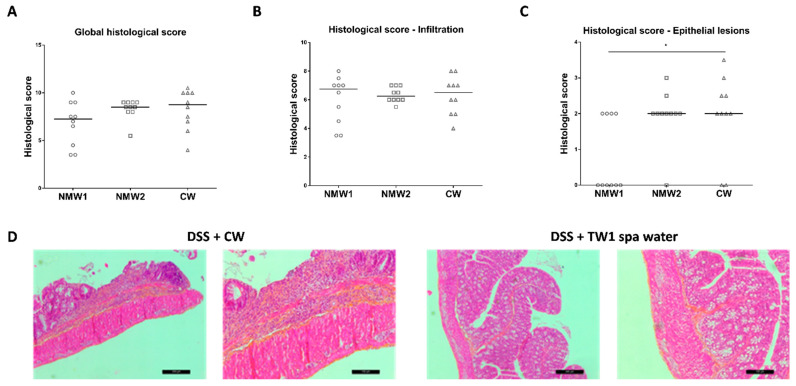
Effect of the administration of NMW1 or NMW2 on histological lesions. (**A**) Global histological score. (**B**) Infiltration of immune cells into the colonic mucosa. (**C**) Evaluation of epithelial lesions in the colonic mucosa. (**D**) HES staining of the colonic mucosa showing epithelial lesions (bar represents 50 µm). The results are presented as the median. Statistical comparisons were carried out after normality testing using Kolmogorov–Smirnov tests, and subsequent one-way ANOVA with the Bonferroni post hoc test was performed (* *p* < 0.05).

At the end of the experiment, frozen colonic tissue sections were prepared for histological assessment of the proximal colon, revealing hemorrhagic walls with multiple ulcerations, mucosal edema, and neutrophil infiltrations with transmural involvement (data not shown). The oral administration of thermal waters from both thermal stations to 1% DSS-treated mice for a 3-week period had no significant impact on the global histological score, taking into consideration both epithelial lesions and immune cell infiltration (Figure 3A and Figure S3). When the impacts of thermal water on immune cell infiltration and epithelial lesions were analyzed separately, we observed that both NMW treatments had no impact on immune cell infiltration (Figure 3B), but the NMW1 treatment efficiently reduced the number of intestinal epithelial lesions induced by 1% DSS in the mouse model (Figure 3C,D). These results demonstrate that NMW1 is more efficient in dampening intestinal inflammation. However, our study suggests that hydrotherapy using NMW is not an appropriate treatment for the active phase of the disease but could be effective in maintaining the quiescent phase of disease.

### 2.3. NMW Treatment Modulates Mucosa-Associated Microbiota in a Mouse Model of Colitis

**Figure 4:**
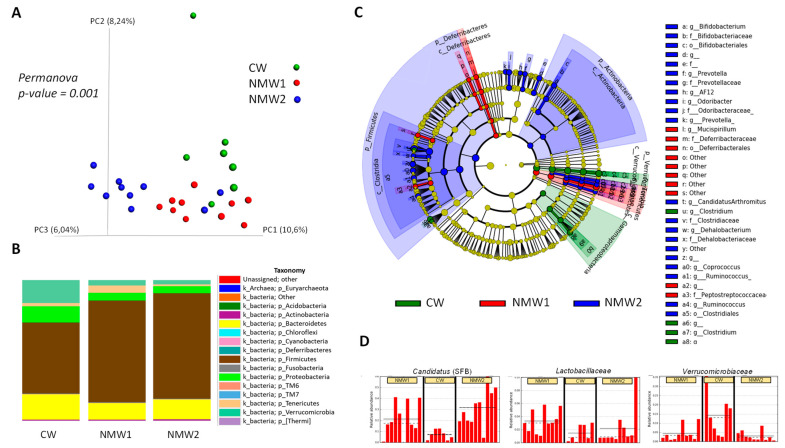
Analysis of the composition of the microbiota associated with the colonic mucosa of mice treated with DSS that received or did not receive NMW1 or NMW2. (**A**) Analysis of beta diversity. (**B**) Comparison of the microbiota at the phylum level. (**C**) Differential analysis of the comparison of the microbiota (LEfSe) between the three conditions. (**D**) Variations in the abundances of different species between the three conditions.

Composition of the colonic mucosa-associated microbiota was evaluated by 16S rRNA gene sequencing in mice treated with 1% DSS. Importantly, treatment with NMW1 and NMW2 led to modified beta diversity (Figure 4A) compared to treatment with regular water. In terms of phyla, treatment with NMW1 and NMW2 led to a higher proportion of Firmicutes and lower percentages of Bacteroidetes and Proteobacteria, a sign of the establishment of a less colitogenic microbiota (Figure 4B). Statistical analysis of differences in the microbiota composition between mice treated or not with NMW1 thermal water using LEfSe analysis demonstrated that NMW1 led to significant increases in *Candidatus arthromitus* (segmented filamentous bacteria, SFB) known to promote Th17 cell differentiation [27] and lactic acid bacteria of the *Lactobacillus* type (Figure 4C,D and Figure S4). Moreover, bacteria belonging to the *Akkermansia* genus, known to have beneficial impacts on metabolic health but a more debatable role in chronic intestinal inflammation [28,29,30], were present in greater abundance in untreated mice than in mice receiving NMW1 (Figure 4C).

Overall, these data indicate that a 3-week treatment with these NMW is sufficient to significantly shift the mucosa-associated microbiota composition toward a more balanced and less colitogenic microbiota. The beneficial effect of particularly NMW1 on IBD with low grade inflammation makes it suitable to investigate the unassessed improvement of human patients with such conditions and the relations of intestinal inflammation improvement and the recovery of a more balanced mucosa-associated microbiota.

## 3. Discussion

In this study, we observed that both NMW1 and NMW2 treatments control the production of the chemoattractant cytokine interleukin-8 produced by intestinal epithelial cells. In addition, NMW1 was more efficient than NMW2 in limiting mild inflammation induced by 1% DSS treatment and enhancing barrier function. Histologically, NMW1 exerted its effect by reducing the number of epithelial lesions, but it did not produce a reduction in immune cell infiltration. This was confirmed by measuring myeloperoxidase (MPO) activity, an enzyme abundant in the cytoplasm of neutrophils (data not shown). Indeed, we observed no decrease in MPO activity after treatment with thermal water. Compared to treatment with control water, treatment with NMW1 or NMW2 produced a reduction in the disease activity index score (DAI score) throughout the treatment period and generated an improvement in the integrity of the intestinal barrier, a reduction in the amount of keratinocyte chemoattractant (KC) released in the colon, a decrease in fecal lipocalin-2 (a marker of intestinal inflammation) expression, and a decreased histological score (especially reduced epithelial damage). However, these beneficial effects were not observed in the 4% DSS-treated model. The 4% DSS model led to a severe inflammation, and the 1% to a moderate inflammation. In view of our results, NMW1 shows beneficial effects when in a context of low-grade or moderate inflammation but cannot treat severe inflammation. Thermal therapy of IBD patients could help control low-grade inflammation during periods of remission, or even increase these periods, but cannot be considered as a treatment for disease flare-ups, existing anti-inflammatory drugs being more effective in this case. The anti-inflammatory effect of thermal waters on DSS colitis models has already been reported. For example, oral treatment with Harkány thermal spring (Hungary) water ameliorates various aspects of DSS-induced colitis in mice, mostly mediated by the hydrogen sulfide content of Harkány water [31]. It has also been reported that ASW (Avene thermal spring water) has immunomodulatory potential. ASW limits the ability of dendritic cells to stimulate Th1- and Th17-type cellular responses by altering their maturation, the production of IL-12 and IL-23, and accessory cellular functions [32]. In addition, the effectiveness of thermal waters from 18 Italian thermal centers was evaluated in 3872 patients with functional dyspepsia and 3609 patients with irritable bowel syndrome, and a significant reduction in the prevalence of symptoms was observed at the end of the first and second cycles of thermal therapy in the dyspeptic and IBS patients [2]. In addition, it has been reported in rat models that administration of deep sea water increased fecal IgA, thus tending to stimulate immune function via a mechanism involving iron mineral [33]. Refined deep-sea water (RDSW), obtained from deep-sea water collected offshore in Muroto (Japan), is mineral-rich drinking water that improves the intestinal environment, increasing fecal SCFAs in human [34].

The microbiota associated with the intestinal mucosa is disturbed in cases of inflammation and is responsible for the chronicity of intestinal disturbances during intestinal inflammation in IBS or IBD patients. NMW seems to impact gut immunity and exert anti-inflammatory properties. Additionally, NMW have the ability to favor a less colitogenic gut microbiota composition, which, in turn, supports the anti-inflammatory milieu already in place. The mechanism of action leading to the beneficial effects of a treatment with NMW1, as well as NMW2, could be the restoration of a more balanced mucosa-associated microbiota, as evidenced by an increase in the abundance of beneficial and immunomodulatory bacteria, such as segmented filamentous bacteria (SFB) or bacteria belonging to the *Lactobacillus* family, and a decrease in the abundance of pathobiont bacteria, as evidenced by the decreases in the Bacteroidetes and Proteobacteria phyla. SFB can induce a healthy state of physiological inflammation in the intestine that protects mice against enteric pathogens [35,36] and participates in establishing long-term protection against intestinal inflammation [37]. The presence of SFB has been detected in the human microbiota where it may contribute, as in mice, to boost mucosal immune responses [38]. Thus, the increase in the abundance of SFB following exposure to NMW1 or NMW2 could partly explain the beneficial effects observed. To our knowledge, this study is the first to show the impact of NMW on the composition of the microbiota associated with the intestinal mucosa in experimental models. Evidence that hot spring waters change the fecal gut microbiota composition is provided by a study on the beneficial effects of consumption of bicarbonate-rich mineral water (BNMW) obtained from the Nagayu hot spring (Taketa, Oita, Japan) in healthy volunteers. BNMW consumption vs. tap water consumption decreased the indexes of glycemic controls, changed the metabolome in blood samples, and demonstrated that the fecal composition of lean-inducible bacteria was increased after BNMW intake [39]. Nevertheless, even if the effect of these particular mineral waters on the intestinal microbiota composition is not yet well established, recent characterizations of the NMW microbiome suggest that NMW could have an impact on bacteria in the gastrointestinal tract [40]. However, changes in the composition of microbial communities after spa treatments are well documented in skin disorders. In patients with moderate to severe forms of psoriasis vulgaris, poor bacterial biodiversity has been noticed, but an increase in *Xanthomonadaceae* family members belonging to the Proteobacteria phylum, which is known to be keratolytic, was associated with clinical improvements observed after a 3-week balneotherapy treatment at a dermatology spa care facility [41]. Patients with atopic dermatitis are predisposed to bacterial superinfection by *Staphylococcus aureus*. Hydrotherapy increases the moisture content of the skin, reduces inflammation in atopic dermatitis lesions, and reduces colonization by *S. aureus* [42]. This could be explained by the antimicrobial effects of acidic hot spring water on *Staphylococcus aureus* strains [43]. Clinical studies have also shown that topics made with this particular NMW increase the Gram-negative bacteria levels with a reduction in Gram-positive bacteria levels and improvements in skin microbial diversity. The concentrations of minerals and nonpathogenic microbes in the NMW of this resort could explain the therapeutic benefit when used to treat inflammatory skin diseases [44].

## 4. Materials and Methods

### 4.1. Natural Mineral Waters

Two natural mineral waters (NMW) were used in the present study: NMW1, a sulphate–carbonic–ferruginous water from the thermal springs of Châtel-Guyon, France; and NMW2, mainly a bicarbonate water from the thermal springs in Vichy, France. The characteristics of these different NMWs are presented in Table 1.

### 4.2. Intestinal Epithelial Cell Culture and IL-8 Measurement

The human intestinal epithelial cell line T84 was obtained from the American Type Culture Collection (ATCC^®^ CCL-248^™^, Manassas, VA, USA) and maintained in an atmosphere containing 5% CO_2_ at 37 °C in the culture medium recommended by ATCC. T84 intestinal epithelial cells were seeded in 24-well tissue culture plates at a density of 2 × 10^5^ cells/well and incubated at 37 °C for 24 h. To induce IL-8 production, T84 cells were treated with an AIEC LF82 bacterial lysate (overnight culture boiled for 5 min) or H_2_O_2_ (1 mM). At the same time, 25 µL or 50 µL of NMW1 and NMW2 or controlled water CW (distilled water) was added, and the IL-8 concentration in the cell supernatant was quantified after a 66-h period. Quantification of the amount of the pro-inflammatory cytokine IL-8 released by human intestinal epithelial cells was performed by ELISA according to the manufacturer’s instructions (R&D Systems, Minneapolis, MN, USA).

### 4.3. Mice and Experimental Protocols

Male Balbc/J mice (body weight, 6 weeks old, approximately 22 g) were purchased from Charles River Laboratories (L’arbresle, France), and the animal care committee of the local government at Clermont Ferrand, France approved all animal procedures. Mice were housed in filter-top cages and provided with food and sterile water ad libitum. After transfer to our animal facility, mice were maintained for 1 week under conventional housing before the beginning of treatment. Wild-type (wt) Balbc/J mice were divided into groups of 10 mice. All groups received either regular tap water or tap water with 1% (*w*/*v*) (to induce moderate inflammation) or 4% (*w*/*v*) (to induce severe inflammation) dextran sulfate sodium (DSS) (M.W. = 36,000–50,000, MP Biomedicals, Illkirch-Graffenstaden, France) as the drinking water. Then, tap water was replaced by CW or NMW1 or NMW2 (still supplemented with 1 or 4% DSS) during a 3-week period. The water was changed every day to preserve the thermal water properties. Animal experiments were performed according to the institutional guidelines approved by the CEMEA Auvergne committee for ethical issues (code 00730.02, 1 September 2013).

### 4.4. Clinical Assessment of Colitis

Animals were observed every day for morbidity, mortality, body weight, stool consistency, and rectal bleeding for the duration of the experiment. Disease activity was evaluated with a clinical scoring system (disease activity index or DAI) assessing weight loss, stool consistency, and rectal bleeding measured by the Hemoccult II test (SKD SARL, Saint-Denis, France), as described in Table 2. This score ranged from 0 (healthy) to 12 (maximal activity of colitis). Mice were sacrificed by cervical dislocation.

### 4.5. Histological Evaluation of Colonic Damage

On day 21, the entire mouse colon was excised, and segments of the proximal colon (1 cm) were fixed in buffered 4% formalin, paraffin embedded, cut into 4-µm sections, and stained with hematoxylin/eosin/safranin (HES). The histological severity of colitis was graded in a “blinded” fashion. The tissue samples were evaluated for the amount and depth of inflammation with a range of 0 to 3 and the amount of crypt damage or regeneration with a range of 0 to 3, as described in Table 3.

### 4.6. Quantification of Fecal Lipocalin-2 and Cytokine Release.

The fecal lipocalin-2 (Lcn-2) concentration was measured by ELISA to detect low-grade inflammation during the course of treatment. Frozen fecal samples were reconstituted in PBS containing 0.1% Tween 20 (100 mg/mL) and processed to obtain a homogenous fecal suspension. The samples were then centrifuged for 10 min at 12,000 rpm and 4 °C. Clear supernatants were collected and stored at −80 °C until analysis. Lcn-2 levels were estimated in the supernatants using a mouse Duoset Lcn-2 ELISA kit (R&D Systems, Minneapolis, MN, USA). Fecal samples were diluted in the kit-recommended reagent diluent (1.0% BSA in PBS, pH 7.2–7.4). Quantification of KC (keratinocyte chemoattractant) pro-inflammatory cytokine release by colonic tissue at the time of sacrifice was performed by ELISA analysis of 50 µL of medium containing released cytokines using kits from R&D Systems following the manufacturer’s instructions.

### 4.7. In Vivo Intestinal Permeability Measurement

In vivo intestinal permeability was measured using FITC-dextran 4 kDa (FD4, Sigma, Saint-Louis, MO, USA). Mice were orally challenged with 15 mg of FD4 diluted in PBS 5 h before blood collection. Serum was collected by centrifugation (30 min, 5500 g), and the FITC concentration was determined by fluorescence measurement and compared with a standard curve of FD4 diluted in serum.

### 4.8. Microbiota Analysis by 16S rRNA Gene Sequencing using Illumina Technology

Genomic DNA from colon samples were lysed in Proteinase K overnight at 56 °C in shaking incubator. DNA were extracted using the Nucleospin^®^ Tissue kit (Macherey-Nagel, Düren, Germany). The DNA concentration was determined with a Qubit^TM^ fluorometer (Invitrogen, Carlsbad, CA, USA), and the DNA quality was evaluated by spectrophotometry (260/280 and 260/230 ratios, NanoDrop™, Thermo Scientific, Waltham, MA, USA). The 16S rRNA genes, region V4, were PCR amplified from each sample using a composite forward primer and a reverse primer containing a unique 12-base barcode, which were designed using the Golay error-correcting scheme and used to tag PCR products from individual samples [45]. We used the forward primer 515F with the sequence 5′-*AATGATACGGCGACCACCGAGATCTACACGCT*XXXXXXXXXXXX**TATGGTAATT*GT***GTGYCAGCMGCCGCGGTAA-3′: the italicized sequence is the 5′ Illumina adapter, the sequence of 12 Xs is the Golay barcode, the bold sequence is the primer pad, the italicized and bold sequence is the primer linker, and the underlined sequence is the conserved bacterial primer 515F. The reverse primer 806R had the sequence 5′-*CAAGCAGAAGACGGCATACGAGAT***AGTCAGCCAG*CC***GGACTACNVGGGTWTCTAAT-3′: the italicized sequence is the 3′ reverse complement sequence of the Illumina adapter, the bold sequence is the primer pad, the italicized and bold sequence is the primer linker, and the underlined sequence is the conserved bacterial primer 806R. PCR mixtures consisted of Hot Master PCR mix (Quantabio, Beverly, MA, USA), 0.2 µM each primer, and 10–100 ng of template, and the reaction conditions were 3 min at 95 °C, followed by 30 cycles of 45 s at 95 °C, 60 s at 50 °C, and 90 s at 72 °C on a BioRad thermocycler. The PCR products were purified with Ampure magnetic purification beads (Agencourt, Brea, CA, USA) and visualized by gel electrophoresis. The products were then quantified using a Quant-iT PicoGreen dsDNA assay (Invitrogen, Carlsbad, CA, USA). A master DNA pool was generated from the purified products mixed in equimolar ratios. The pooled products were quantified using the Quant-iT PicoGreen dsDNA assay and then sequenced using an Illumina MiSeq sequencer (paired-end reads, 2 × 250 bp) at Cornell University, Ithaca.

### 4.9. 16S rRNA Gene Sequencing Analysis

Forward and reverse Illumina reads were joined using the fastq-join method, and sequences were demultiplexed and quality filtered using the Quantitative Insights Into Microbial Ecology (QIIME, version 1.8.0) software package [46]. QIIME default parameters were used for quality filtering (reads truncated at the first low-quality base and excluded if (1) there were more than three consecutive low-quality base calls, (2) less than 75% of the read length was consecutive high-quality base calls, (3) at least one uncalled base was present, (4) more than 1.5 errors were present in the barcode, (5) any Phred qualities were below 20, or (6) the length was less than 75 bases). Sequences were assigned to operational taxonomic units (OTUs) using the UCLUST algorithm [47] with a 97% threshold for pairwise identity (with the creation of new clusters with sequences that did not match the reference sequences) and classified taxonomically using the Greengenes reference database 13_8 [48]. A single representative sequence for each OTU was aligned, and a phylogenetic tree was built using FastTree [49]. The phylogenetic tree was used for computing the unweighted UniFrac distances between samples [50,51], and rarefaction was performed and used to compare the abundances of OTUs across samples. Principal coordinates analysis (PCoA) plots were used to assess the variation between experimental groups (beta diversity).

### 4.10. Statistical Analysis

Statistical analyses were performed using the GraphPad Prism V.7.0 (GraphPad Software, San Diego, CA, USA) software package for PC. For all data displayed in graphs, values are expressed as the mean ± SEM or median. Data comparisons between two groups were performed using a two-tailed Student’s *t*-test analysis or a Mann–Whitney U-test depending on data normality determined using the Kolmogorov–Smirnov test. When appropriate, a one-way ANOVA test with the Bonferroni post hoc test was performed. Differences corresponding to *p* values ≤ 0.05 were considered statistically significant. Differences in microbiota composition between groups were investigated by Permanova approach using the compare_categories tool through Qiime.

## 5. Conclusions

To conclude, this study showed that both tested NMW from Châtel-Guyon (NMW1) and Vichy (NMW2) exhibited anti-inflammatory properties in *in vitro* and *in vivo* models, more importantly with NMW1. These anti-inflammatory effects could be mediated by rebalancing the mucosa-associated microbiota composition, which is usually destabilized during inflammation. However, our work also showed that this treatment is less effective in severe colitis models, suggesting less relevance for the management of active disease. Thus, spa treatment could be discussed to maintain a state of low inflammation rather than to treat active diseases needing more effective pharmacological treatments. Thus, the use of spa treatment as a complementary therapy to extend periods of “low grade” inflammation in IBD patients should be investigated by relevant clinical investigation, assessing the impact of spa treatment on the composition of the intestinal microbiota and determining the relations between patients’ improvement and rebalancing the intestinal microbiota.

## Figures and Tables

**Table 1:** Physical and chemical properties of the two natural mineral waters used in this study (**NMW1**: F3 Spring, Châtel-Guyon, Puy de Dôme, France; **NMW2**: Antoine and Boussange Springs, Vichy, Allier, France).

	NMW1	NMW2
T°C	37	36
pH	6.4	6.8
		
Mineral elements content (mg/L)		
Total content	6495	4687
CO2 free	3062	759.9
H2S	<0.10	0
Sodium	900	1670
Calcium	515	67.7
Magnesium	311	8.9
Potassium	74.8	83.8
Iron	15.1	0.461
Silicon	110.5	73.8
Lithium	4.67	4.6
Bromine	5.1	1.25
Manganese	0.24	0.072
Sulphate	326	168.9
Chlorine	2111	294.5
Bicarbonate	0	4232.3
Fluor	0.5	10.78
Strontium	8.97	0.461
Aluminum	<0.001	0.034
Arsenic	0.083	0.602
Boron	1.52	2.1

**Table 2:** Disease activity index (DAI) assessment.

	Score	Characteristic(s)
Body weight loss	0	No loss
	1	1 to 5% loss of body weight
	2	5 to 10% loss of body weight
	3	10 to 20% loss of body weight
	4	>20% loss of body weight
Stool consistency	0	normal feces
	1	loose stool
	2	watery diarrhea
	3	slimy diarrhea, little blood
	4	severe watery diarrhea with blood
Blood in stool	0	no blood
	2	presence of blood assessed by Hemoccult II test
	4	visible bleeding

**Table 3:** Histological grading of intestinal inflammation.

	Score	Characteristic(s)
Infiltration of inflammatory	0	rare inflammatory cells in the lamina propria
Cells	1	increased numbers of inflammatory cells, including neutrophils in the lamina propria
	2	confluence of inflammatory cells extending into the submucosa
	3	transmural extension of the inflammatory cell infiltrate
Infiltration of lamina propria	0	normal
by mononuclear cells	1	moderate
	2	great
Infiltration of lamina propria	0	normal
by polynuclear cells	1	moderate
	2	great
Infiltration of epithelium	0	no infiltration
by polynuclear cells	1	surface
	2	inside the crypt
	3	cryptic abscess
Severity of epithelial damage	0	absence of mucosal damage
	1	lymphoepithelial lesions
	2	mucosal erosion/ulceration
	3	extensive mucosal damage and extension through deeper structures of the bowel wall
Surface of epithelial damage	0	normal
	1	focal
	2	wide

## Data Availability

Data available on request. The data underlying this article will be shared on reasonable request to the corresponding author.

## Supplementary Materials

The following are available online at https://www.mdpi.com/article/10.3390/ijms22094336/s1, Figure S1: Effet of NMW treatment on the intestinal barrier in vivo. Intestinal permeability was measured in NMW or CW mice treated with 1% DSS. The results are expressed in µg of FD4/mL of serum 5 h after intragastric administration, Figure S2: Effect of the administration of NMW 1 or NMW2 on body weight and DAI in a 4% DSS-induced inflammation model, Figure S3: Effect of the administration of NMW1 or NMW2 on histological lesions. HES staining of the colonic mucosa from five different mice (NT: non treated; treated 1 %DSS, treated 1% DSS + NMW1; treated 1% DSS + NMW2). Bar represents 50 µm, Figure S4: Analysis of the composition of the microbiota associated with the colonic mucosa of mice treated with DSS that received or did not receive NMW1 or NMW2. Differential analysis of the comparison of the microbiota (LEfSe) between the three conditions.
