# Supplementary material for: Beneficial Effects of Natural Mineral Waters on Intestinal Inflammation and the Mucosa-Associated Microbiota
Source: Int J Mol Sci. 2021 Apr 21;22(9):4336. doi: 10.3390/ijms22094336 (PMC8122343; doi:10.3390/ijms22094336)
Supplement: Supplementary file 1 [file ijms-22-04336-s001.zip › ijms-1167619-supplementary.pdf]

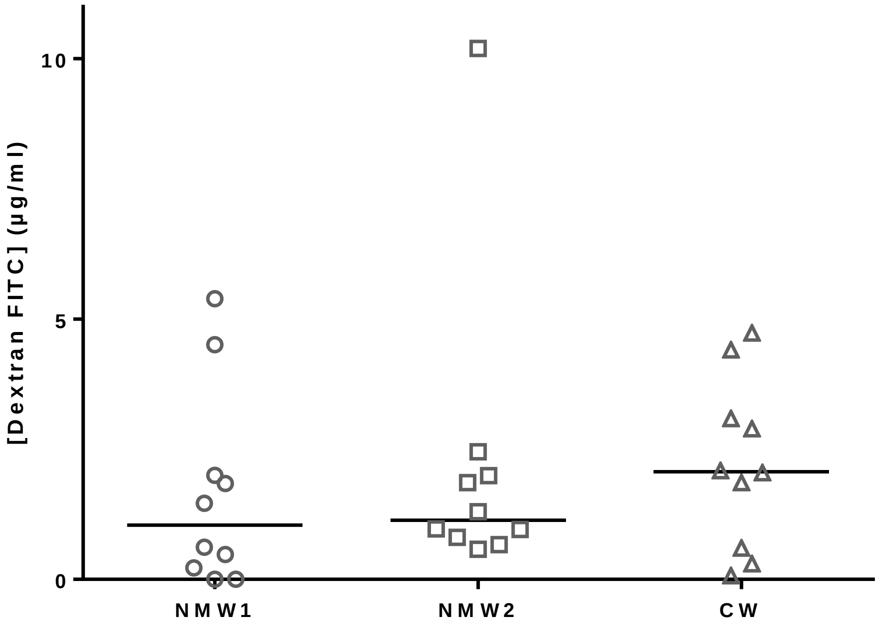

Figure S1. Effect of NMW treatment on the intestinal barrier *in vivo*. Intestinal permeability was measured in NMW or CW mice treated with 1% DSS. The results are expressed in µg of FD4/mL of serum 5 h after intragastric administration.

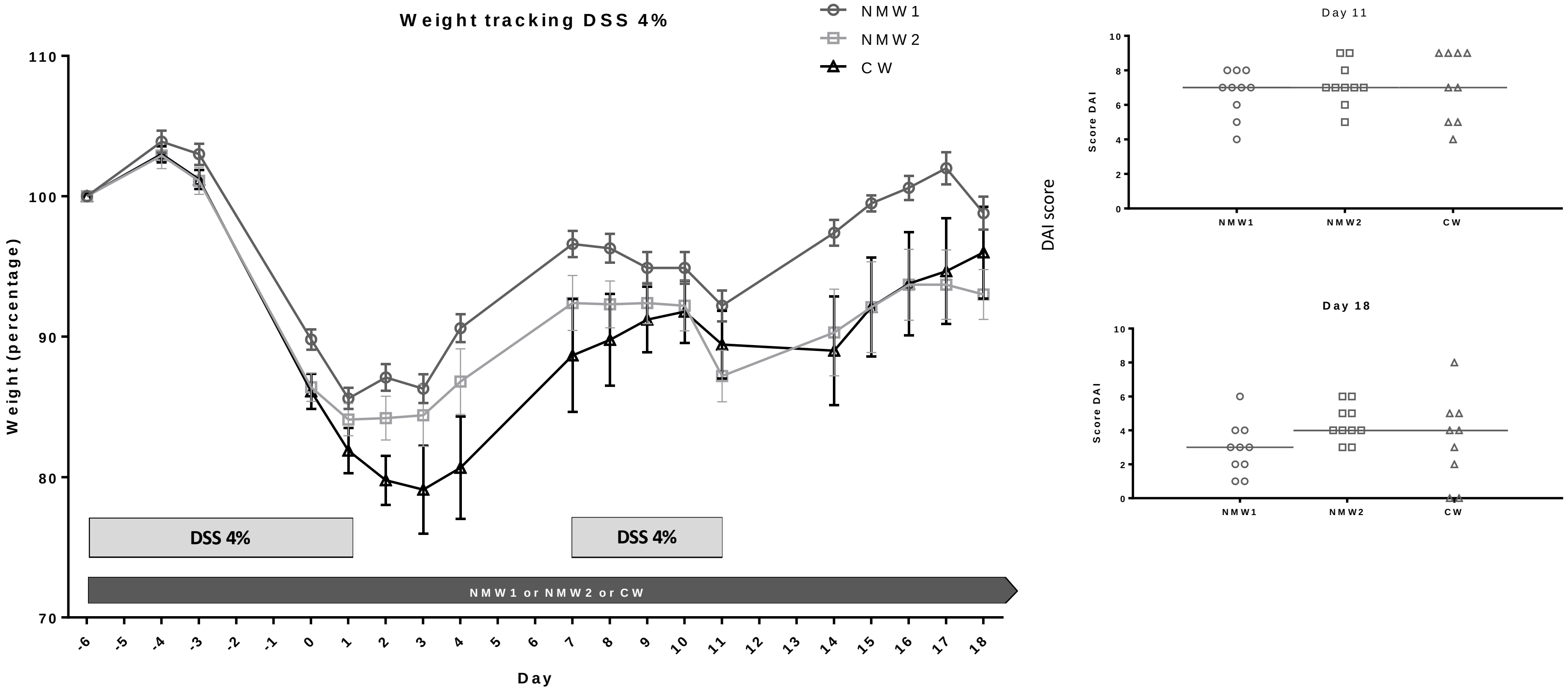

Figure S2. Effect of the administration of NMW 1 or NMW2 on body weight and DAI in a 4% DSS-induced inflammation model.

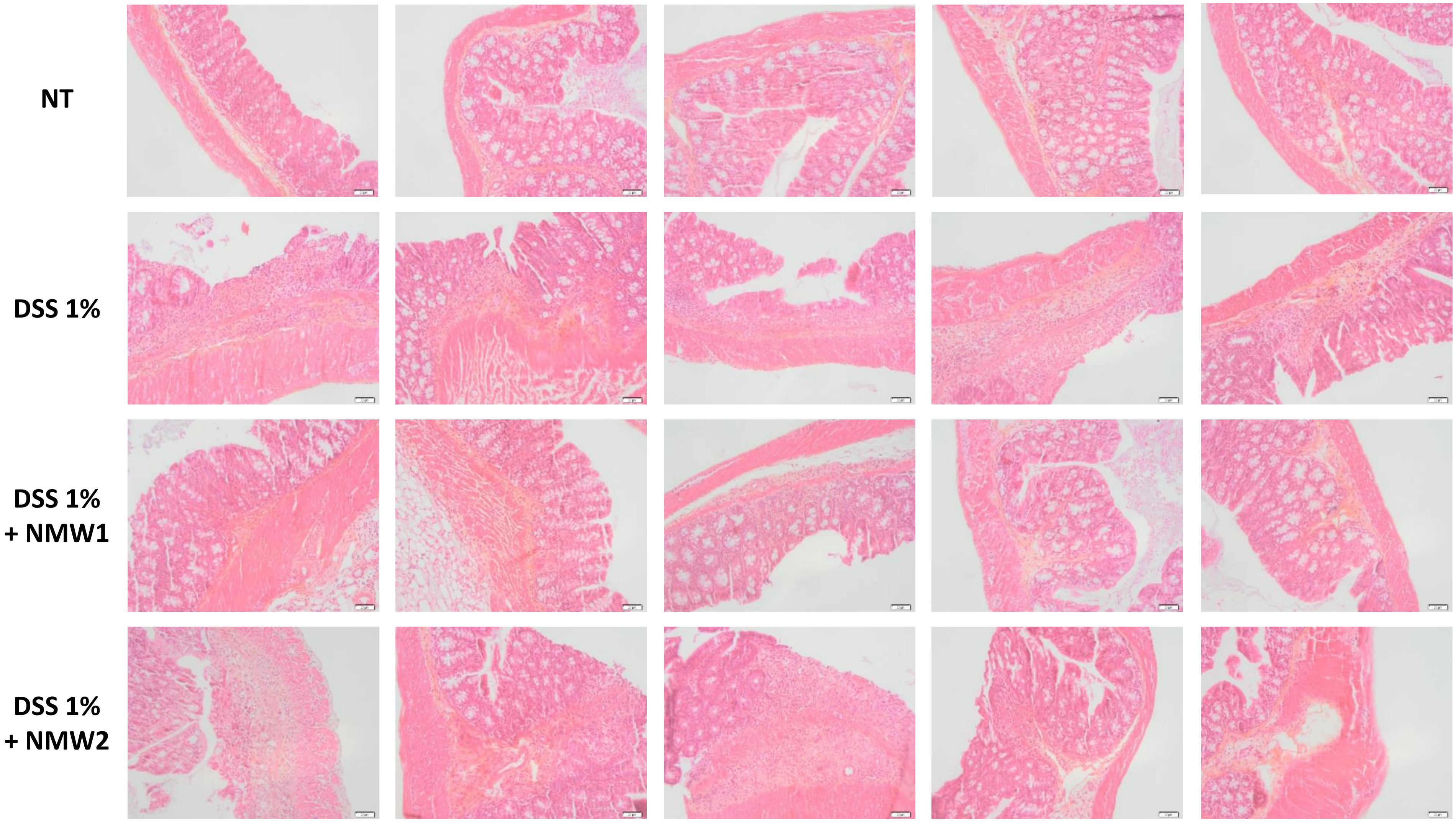

Figure S3. Effect of the administration of NMW1 or NMW2 on histological lesions. HES staining of the colonic mucosa from 5 different mice (NT: non treated; treated 1 %DSS, treated 1% DSS + NMW1; treated 1% DSS + NMW2). Bar represents 50 μm.

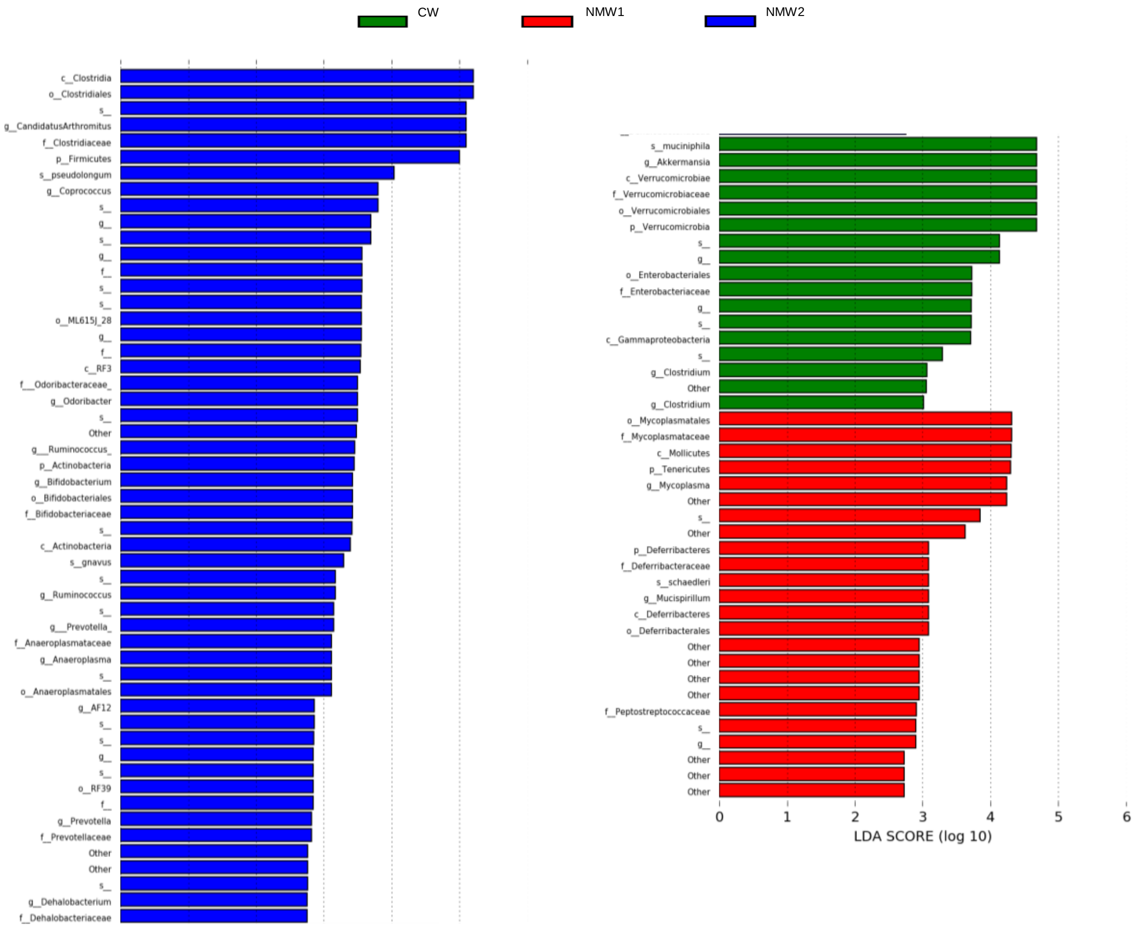

Figure S4. Analysis of the composition of the microbiota associated with the colonic mucosa of mice treated with DSS that received or did not receive NMW1 or NMW2. Differential analysis of the comparison of the microbiota (LEfSe) between the three conditions.
